# Simple Culture Methods and Treatment to Study Hormonal Regulation of Ovule Development

**DOI:** 10.3389/fpls.2018.00784

**Published:** 2018-06-18

**Authors:** Bu-Fan Li, Shi-Xia Yu, Li-Qin Hu, Yan-Jie Zhang, Ning Zhai, Lin Xu, Wen-Hui Lin

**Affiliations:** ^1^Joint International Research Laboratory of Metabolic & Developmental Sciences, School of Life Sciences & Biotechnology, Shanghai Jiao Tong University, Shanghai, China; ^2^National Key Laboratory of Plant Molecular Genetics, CAS Center for Excellence in Molecular Plant Sciences, Institute of Plant Physiology and Ecology, Chinese Academy of Sciences, Shanghai, China; ^3^University of Chinese Academy of Sciences, Beijing, China

**Keywords:** ovule development, phytohormones, treatment in living plant, culture medium for excised flower/pistil, brassinosteroids

## Abstract

Ovule development is one of the most important processes in the reproductive development of higher plants and is a determinant of seed quality and quantity. Phytohormones play key roles in this process since loss-of-function mutants in hormone signaling show defective ovule phenotypes and reduced fertility. However, it is difficult to distinguish the direct effects of hormones on ovule development because it is parts of reproductive development and the defective phenotypes would be the indirect effects following the defective vegetative development. The treatment of hormones is a direct method to investigate the hormonal regulation of ovule development, but ovule is embedded inside several layers of floral organs, and traditional methods for hormone (or inhibitor) treatments have various limitations. We have developed simple methods to apply treatments to the flowers in a living plant, where an inflorescence apex is immersed into a solution in an inverted tube. We have also developed a specific system to culture and treat excised flowers/pistils. These procedures will be useful for research on the hormonal regulation of ovule development. We provide examples of how treatments with brassinosteroids (BR) and BR biosynthesis inhibitor. We cultured and treated plant materials using our newly developed methods, and observed the morphology of wild type ovules and fluorescence signals in a marker line to monitor the progress of ovule development. The results demonstrate BR promotes ovule development and our new methods are efficient and repeatable.

## Introduction

The ovule harbors the female gametophyte and is the precursor of seeds. Ovule development is one of the most important processes in plant reproductive development and it plays essential roles in determining seed quality and quantity. Therefore, research on the ovule is critical for the seed industry to generate higher-yielding plants. However, it is difficult to study ovule development because the ovule is deeply embedded in flowers and is protected by several layers of tissues and organs.

The developmental stage of the ovule cannot be directly observed from the surface, but may be estimated from the features of the floral organs. Ovule development can be divided several main steps as follows (Robles and Pelaz, 2005; Roeder and Yanofsky, 2006): First, the carpel margin meristem develops into the placenta, septum, and transmitting track. This stage corresponds to stage 8 of flower development (Bowman et al., 1999; Ferrandiz et al., 1999; Ferrándiz et al., 2010). Second, the ovule primordia initiate from the placenta (stage 9, Smyth et al., 1990; Roeder and Yanofsky, 2006). Third, three different regions (the funiculus, the chalaza, and the nucellus) become organized along the proximal-distal axis (stage 10, Gasser et al., 1998; Skinner et al., 2004). Fourth, within the nucellus, the megaspore mother cell undergoes meiosis to generate four megaspores, one of which is the functional megaspore (stage 11, Smyth et al., 1990; Shi and Yang, 2011). Finally, the functional megaspore undergoes three rounds of mitosis to generate the mature haploid embryo sac containing seven cells with eight nuclei (stage 12, Smyth et al., 1990; Chaudhury et al., 1998; Cucinotta et al., 2014; Schmid et al., 2015). The descriptions of the stages of ovule and flower development vary in the literature (Galbiati et al., 2013), but the ovule developmental process is the same. If any of these morphogenetic processes malfunctions, the ovule may fail to develop or die.

Ovule development is regulated by hormones (Nole-Wilson et al., 2010; Bartrina et al., 2011; Bencivenga et al., 2012; Marsch-Martinez et al., 2012; Huang et al., 2013), and environmental factors (Sun et al., 2004; Su et al., 2013). These conclusions have been derived from analyses of mutant phenotypes, rather than from direct physiological analyses. In the model plant Arabidopsis, ovules are coated in the pistil, which is encased in the flower and surrounded by outer organs such as stamens, petals, and sepals (Smyth et al., 1990). Because the ovules are hidden, it is difficult to apply exogenous substances such as hormones or other chemicals such as inhibitors or nutrients. In experiments on roots or seedlings, the effects of substances such as hormones can be determined by adding them to the medium (Ogawa et al., 2003; Yoshimitsu et al., 2011) or by briefly dipping the root or seedling into a hydroponic solution containing the substance of interest (D’Aloia et al., 2011; Cucinotta et al., 2016). For experiments on reproductive organs, spraying is the most popular method of applying hormones or other substances. The solution containing the compound of interest is sprayed directly onto the surface of plant tissue until it forms a uniform mist layer (Zuniga-Mayo et al., 2014; Zhang et al., 2016).

*In vitro* cultures of plant tissues have been intensively studied, and are easily produced due to the totipotency of plant cells (Horstman et al., 2017; Melnyk, 2017). Different proportions of plant hormones in the medium can induce explants to grow roots or shoots, which subsequently develop into new plants (Huang and Yeoman, 1984; Bassuner et al., 2007; Cheng et al., 2010; Gaj, 2011). Most *in vitro* cultures of reproductive organs have focused on pollen (Johnson-Brousseau and Mccormick, 2004; Palanivelu and Preuss, 2006). Such studies have shown that non-pollinated endosperm can spontaneously generate without fertilization (Rojek et al., 2005), and that freshly fertilized ovules can de-differentiate and eventually develop into whole plants (Sauer and Friml, 2004). The excised fertilized pistils are shortly cultured to study pollen tube rupture (Duan et al., 2013).

Although *in vitro* ovule cultures would be useful to study the roles of hormones in ovule development, few high-efficiency systems are available for such analyses. Here, we develop simple culture systems for excised flower/pistil which allows ovules to initiate and develop normally inside excised flower/pistil and we can apply exogenous hormones and observe hormonal regulation directly. Also we develop the treatment system for living plants which makes it possible to conduct stable and repeatable hormonal/chemical treatments of flowers on intact plants. The morphological analysis of Arabidopsis *Col-0* ovules and the fluorescence signal observation of *pKNU: KNU-Venus* marker line (a marker line labeled megaspore mother cell identity in *Ler* background, Payne et al., 2004) demonstrated we obtained reliable results by applying hormones and inhibitors using our methods, indicating that these systems are suitable for research on the hormonal (chemical) regulation of ovule development.

## Materials and Methods

### Plant Material and Growth Conditions

Seeds of *Col-0* and the *pKNU:KNU-Venus* marker line (*Ler* background) were surface-sterilized with 75% (v/v) ethanol for 10 min, washed five times with sterilized water, and then sown on 1/2 MS (Murashige and Skoog) solid medium (0.8% w/v agar). The plates were incubated in the dark at 4°C for 3 days and then the seedlings were grown under 16-h light/8-h dark photoperiod at 22°C. Seven-day-old seedlings were transferred to a mixed soil medium (vermiculite: nutritive soil: perlite = 10:10:1) and grown under a 16-h light/8-h photoperiod at 20–22°C.

### Treatment Method for Living Plants

To treat flowers in a living plant, a 200 μL PCR tube was attached upside-down on a stick, and 50–100 μL hormone solution diluted with ddH_2_O or 0.02% Tween 20 (Sangon Biotech A600560) or 0.01% Triton^TM^ X-100 (Sigma T9284) was added to the bottom of the tube. After removing flowers older than a certain stage (depending on which developmental process was being observed) or marking the flowers targeted for treatment, the inflorescence apex was immersed in the hormone solution for 6–8 h. The tube with the solution was removed after the treatment, and the ovules from the treated flowers were observed after 1–3 days, depending on which materials were treated and which developmental process was being observed (it took longer for the ovule to develop from stage 9 to 10 than from stage 8 to 9). The flowers of the *pKNU:KNU-Venus* marker line (*Ler* background) grew slower than those of *Col-0*.

### Culture Medium for Excised Flowers/Pistils

The culture medium was modified from ICM and AM medium (Sauer and Friml, 2004), and consisted of 1/2 MS with 10% (w/v) sucrose (Sigma P1888) and 0.05% (v/v) MES (Amresco, Solon, OH, United States). The pH was adjusted to 5.8 with 1 mol/L KOH before adding 0.5% (w/v) phytagel (Sigma P8169; Sigma, St. Louis, MO, United States). After autoclaving at 120°C for 20 min, the medium was cooled and glutamine was added to a final concentration of 400 mg/L. The medium was then poured into clean Petri dishes (3.5 cm). The prepared medium could be stored at 4°C for a short time but needed to be used as soon as possible.

### Preparation of Materials for Culture Systems

**FIGURE 1:**
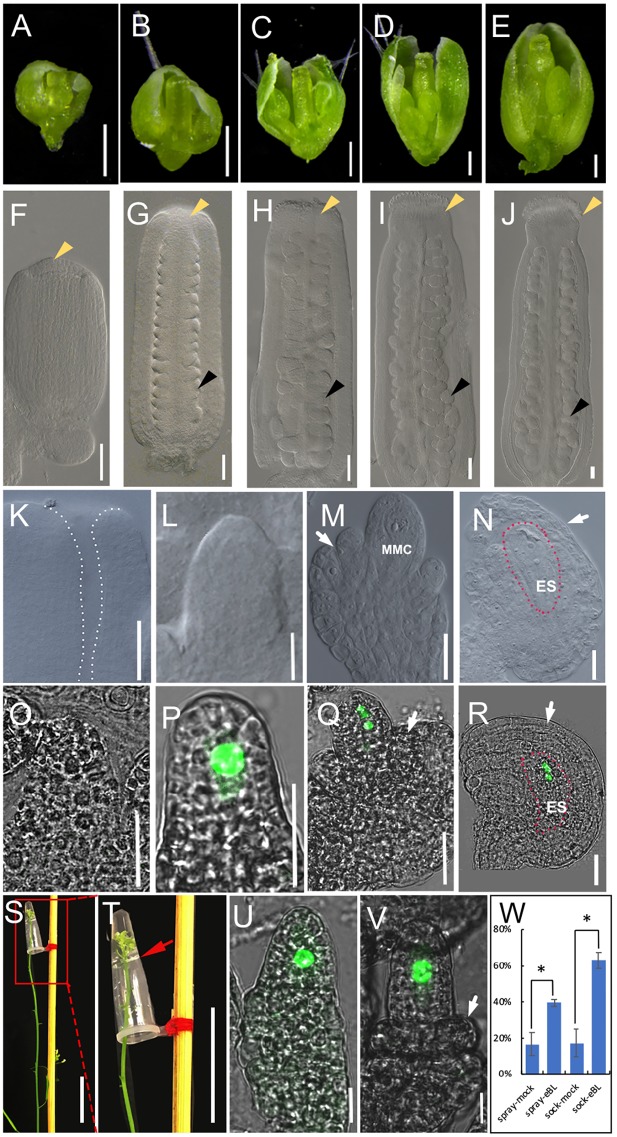
The development of pistils and ovules in living plant and BR treatment in living plant. **(A–E)** The development of flowers in living plant at flower developmental stage 8 **(A)**, stage 9 **(B)**, stage 10 **(C)**, stage 11 **(D)**, and stage12 **(E)**. **(F–J)** The development of pistils in living plant at flower developmental stage 8 **(F)**, stage 9 **(G)**, stage 10 **(H)**, stage 11 **(I)**, and stage12 **(J)**. Yellow arrowheads indicate the stigmas, and black arrowheads indicate example ovules. **(K–N)** The development of ovules in living plant at flower developmental stage 8 (**K**, no ovule initiation), stage 10 **(L)**, stage 11 **(M)**, and stage12 **(N)**. The area highlighted by the dotted line indicate the flat placenta **(K)** and the embryo sac in **(N)**. **(O–R)** The fluorescence signal *of pKNU:KNU-Venus* in living plant at flower developmental stage 9 (**O**, no signal), stage 10 **(P)**, stage 11 **(Q)**, and stage 12 **(R)**. The areas highlighted by the dotted line indicate the embryo sac **(R)**. **(S,T)** Treatment method in living plant. We remove older flowers in inflorescence apex. **(T)** The magnification of the red area of **(O)**. The red arrow indicates liquid surface. **(U,V)** The fluorescence signal in the nuclear of MMC in mock **(U)** and 1 × 10^-8^ mol/L eBL solution **(V)** after hormonal treatment for 3 days. **(W)** Statistics analysis of ovule development after 1 × 10^-8^ mol/L eBL treatment in living flower. The student *t*-test was used to analyze the significant differences between different treatment method (^∗^*p* < 0.05, ^∗∗^*p* < 0.01). MMC, macrospore mother cell; ES, embryo sac. White arrows indicate the integuments in **(M,N,Q,R,V)**. Bars, **A–E**, 200 μm; **F–R,U,V**, 20 μm; **S,T**, 2 cm.

Flowers in stage 8 and early stage 9 were selected under a dissecting microscope (DFC450, Leica Microsystems, Wetzlar, Germany) located inside a clean hood. The developmental stages were identified according to the morphology of petals, pistils, and stamens. In the flowers in stage 8, the petals had not yet appeared, and the pistil length was about 100 μm (**Figure 1A**). In the flowers in early stage 9, the petals were visible but were not longer than the stamens, and the pistils were longer than the stamens (pistil length approx. 150 μm) (**Figure 1B**; Smyth et al., 1990). We developed two culture systems, one for flowers and one for pistils. In the flower culture system, the flower stalks were inserted into the medium so that the whole flowers were vertical. In the pistil culture system, the sepals, petals, and stamens were removed or uncovered, and the pistils were laid down on the medium horizontally. The open Petri dish (3.5 cm) containing medium and pistils was placed in a larger closed dish (10 cm) containing a piece of wet and sterilized filter paper to create 100% humidity conditions (**Figure 3A**, modified from Johnson-Brousseau and Mccormick, 2004). In both methods, the dishes were incubated under a 16-h light/8-h dark photoperiod at 22°C.

### Hormone and Inhibitor Treatments in Culture Systems

In the flower culture method, 1 × 10^-9^, 1 × 10^-8^, 1 × 10^-7^ mol/L 24-epibrassinolide (eBL) or 1 × 10^-6^mol/L brassinazole (BRZ, a brassinosteroids biosynthesis inhibitor) were each added directly to the medium. The excised flowers were pushed into the medium for culturing as previously described. In the pistil culture method, 1 μL 1 × 10^-8^ mol/L eBL or 1 × 10^-6^mol/L BRZ solution was dropped directly onto a single excised pistil with a pipette. The pistils were immersed in, and slowly absorbed, the hormone solution.

### Observation of Ovules by Differential Interference Microscopy and Confocal Laser Microscopy

The samples used for these analyses were fresh flowers/pistils of *Col-0* and the marker line, excised pistils/flowers after 7–10 days of growth in the culture system, or flowers/pistils after hormone/chemical treatments in living plants or in the culture system. Samples were treated with Hoyer’s solution (chloral hydrate: glycerol: water = 8:1:3, w/v/v) for 2 h. The sepals, petals, and stamens were removed from flowers, the pistils were cut open along the replum with a needle, and the ovules were carefully transferred to a glass slide under a dissecting scope. The samples were then observed under a differential interference microscope (DIC, Axio Imager M2, Zeiss, Göttingen, Germany). The ovules of *pKNU:KNU-Venus* were observed under a confocal laser microscope (TCS SP8, Leica Microsystems).

## Results

### Treatment of Living Plants

Before beginning our research on the hormonal regulation of ovule development and improving the treatment systems, we observed flower, pistil and ovule morphology to distinguish the developmental characters of flowers and ovules at different developmental stages. We monitored the appearance of sepals, petals, and anthers, pistil size (**Figures 1A–E**), and the stage of stigma development (**Figures 1F–J**) to judge the developmental stage of the flower and ovule. Based on our observations, the ovule primordia had not initiated from the placenta in stage 8 of flower development (**Figure 1K**). In stage 9 (**Figure 1G**), ovule primordia had initiated and grown into a finger-like shape. The funiculus, the chalaza, and the nucellus were organized along the proximal–distal axis in stage 10 (**Figure 1L**). The megaspore mother cell and a functional megaspore were present in stage 11 (**Figure 1M**) and megagametogenesis occurred in stage 12 (**Figure 1N**). These morphologies in intact plant are the controls of hormones treatments in living plant, the ovule growth and development in excised tissues, and hormones treatments of excised tissues.

We also observed ovule development in the *pKNU:KNU-Venus* marker line. The fluorescence signal of KNU-Venus was not observed in stage 9 (**Figure 1O**), but was strong in the megaspore mother cell in stage 10 (**Figure 1P**). The signal was evident during meiosis in stage 11 (**Figure 1Q**) and the mitotic events during embryo sac formation in stage 12 (**Figure 1R**; Payne et al., 2004). These patterns of fluorescence signal are the controls of hormones treatments in living plant, the ovule growth and development in excised tissues, and hormones treatments of excised tissues.

Although hormone molecules are quite small, it is difficult for them to enter the tissues of adult plants. We tried to enhance the repeatability and stability of hormone treatments by using a modified dipping method, similar to that used in Arabidopsis transformation methods. The traditional dipping method was not suitable for long-term treatments and it had the potential to cause physical and gravitational stress. We modified the dipping method as described below and gained satisfactory results after treating the living Arabidopsis inflorescence apex with hormones (**Figures 1S,T**). A small volume (50–100 μL) of hormone solution was placed in a 200-μL PCR tube, and the tube was fixed upside-down on a stick. Due to the cohesion among water molecules in the solution and the tension between water molecules and the inner wall of the tube, the hormone solution did not flow out by gravity. We removed the older flowers (or marked the older flowers that would not be treated) and marked the youngest flower buds, and then immersed the inflorescence apex with flower buds into the hormone solution in the tube for 6–8 h. The treatment was stable and well controlled by time and hormone concentration. After the treatment, we removed the hormone solution and allowed the treated inflorescence and flowers continue to grow normally for 1–3 days before observations (depending on which developmental process was being observed, it took longer for the ovule to develop from stage 9 to 10 than from stage 8 to 9). Plants were treated with a mock solution and kept under the same growth conditions as the control to account for the slight stress of soaking.

To test the efficiency of our method, we conducted experiments in which we applied BR (eBL) to the inflorescence apex. BR has been shown to promote ovule development (Nole-Wilson et al., 2010; Pérez-España et al., 2011; Huang et al., 2013). We used our method to test the effects of eBL at different concentrations on ovule development, and found that 1 × 10^-8^ mol/L eBL had the highest efficiency in promoting ovule development (Supplementary Figure S5A). Therefore, we used this concentration in further experiments. Older flowers (later than stage 10) were removed from the inflorescence apex of *pKNU:KNU-Venus*, and the remaining youngest buds were marked. The inflorescence apexes with the buds were soaked in the eBL solution or the mock solution for 8 h, and then grew for a further 3 days. The treated flowers were observed by confocal laser microscopy. We found that the ovules from 63.17% flowers had initiating integuments after the eBL treatment, compared with 17.23% after treatment with the mock solution, demonstrating that eBL promotes ovule development. When eBL was applied by spraying, the ovules from 39.53% flowers had initiating integuments after 3 days, compared with 16.57% of those sprayed with the mock solution. These results indicated that both two methods demonstrated BR promoted ovule development and our method to apply hormone treatments to living plant showed more clear results comparing to the spray method (**Figures 1U–W**).

### Normal Growth of Excised Flowers on Culture Medium

Besides treating flowers on a living plant, we further developed two methods to treat excised flowers and pistils. These systems will be useful for conducting physical treatments with various hormones and chemicals because it is easier to identify flowers at the same stage to conduct parallel experiments.

First, we cultured excised young pistils and flowers on 1/2 MS medium. On the basis of our observations, we concluded that the excised pistils in stage 8 could not grow on 1/2 MS medium, only some ovules could initiate, but the initiated ovules were not able to develop to later developmental stages (Supplementary Figures S1A,B). A few excised pistils in stage 9 were able to grow slightly, but the initiated ovule could not develop to the next stage (Supplementary Figures S1C,D). The excised flowers in stage 8 could not develop to stage 11 on 1/2 MS medium (Supplementary Figures S2A–D), although a small proportion of excised flowers in stage 9 could develop to stage 12 (Supplementary Figures S2E–J). To improve the growth and development of the excised flowers/pistils, we modified the growth medium. The new medium consisted of 1/2 MS, 10% (w/v) sucrose, 0.05% (v/v) MES, 0.5% (w/v) phytagel, and 400 mg/L glutamine (pH = 5.8, detailed description in methods). This medium was a modified medium of the ICM and AM medium that has been used previously for the *in vitro* culture of Arabidopsis fertilized ovules and embryos (Sauer and Friml, 2004).

**FIGURE 2:**
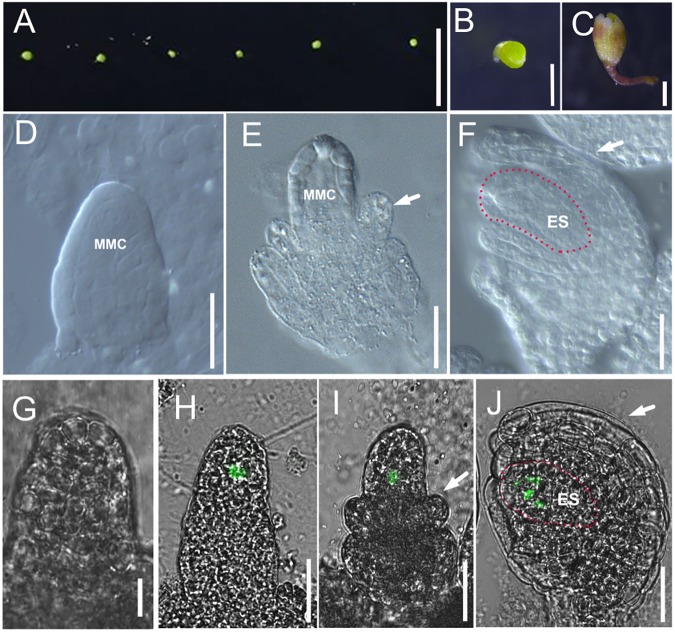
Culture method for excised flower and ovule development in cultured flowers. **(A,B)** Flower buds at flower developmental stage 8 are plugged into culture medium. **(C)** Flowers after 7 days growing in modified culture medium. **(D–F)** DIC observation of ovules inside cultured Col-0 flowers at stage 10 **(D)**, stage 11 **(E)**, and stage 12 **(F)**. White arrows in **(E,F)** indicate the integuments, and the areas highlighted by the dotted line indicate the embryo sac in **(F)**. **(G–J)** The fluorescence signal in the ovules *of pKNU:KNU-Venus* insides the cultured flowers at stage 9 (**G**, no signal), stage 10 **(H)**, stage 11 **(I)**, and stage 12 **(J)**. White arrows in **(I,J)** indicate the integuments, and the areas highlighted by the dotted line indicate the embryo sac in **(J)**. MMC, macrospore mother cell; ES, embryo sac. Bars, **A**, 5 mm; **B,C**, 500 μm; **D–J**, 20 μm.

We started culturing excised flowers (**Figures 2A,B**) from stage 8 to study ovule primordia initiation, because this process is the main determinant of ovule number. We also cultured flowers from stage 9 to study nucellus/megaspore mother cell development and integument initiation. After 7 days of culture (**Figure 2C**), the ovules from 16.67% excised flowers in stage 8 had developed to late stage 11 (11.11%) and stage 12 (5.56%, integuments beginning to envelop the nucellus) on our medium (**Figures 2D–F**). We observed the fluorescence signals in the megaspore mother cells, megaspore cells, and embryo sac of the *pKNU:KNU-Venus* marker line (**Figures 2G–J**). The results indicated that ovules developed normally and functional female cells were produced in this system. We found that the ovules from all excised flowers in stage 9 developed pasted late stage 11 (Supplementary Figures S3A–F). These results indicated that the flowers could grow, the ovules could initiate and develop, the megaspore mother cell could differentiate, and the megaspore cells and embryo sac could develop on our medium. As expected, the later the developmental stage of the excised flower, the better the growth on the modified medium.

### Normal Growth of Excised Pistils in Culture System

**FIGURE 3:**
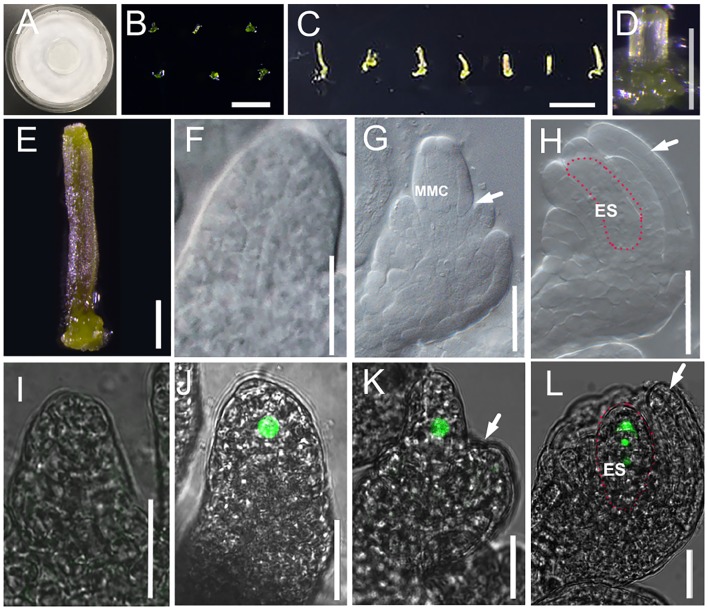
Culture system for excised pistil and ovule development in cultured pistils. **(A)** Culture system for excised pistil; **(B–E)** Pistils at flower developmental stage 8 are laid down onto culture medium after removing other flower organs **(B)** and after 7 days growing **(C)**. Single pistil before **(D)** and after **(E)** 7 days growing. **(F–H)** DIC observation of ovules inside cultured *Col-0* pistils at stage 10 **(F)**, stage 11 **(G)**, and stage 12 **(H)**. White arrows in **(G)** and **(H)** indicate the integuments, and areas highlighted by the dotted line indicate the embryo sac in **(H)**. **(I–L)** The fluorescence signal in the ovules of *pKNU:KNU-Venus* insides the cultured pistils at stage 9 (**I**, no signal), stage 10 **(J)**, stage 11 **(K)**, and stage 12 **(L)**. White arrows in **(J,K,H,L)** indicate the integuments, and the areas highlighted by the dotted line indicate the embryo sac in **(H,K)**. MMC, macrospore mother cell; ES: embryo sac. Bars, **B**, 1 mm; **C**, 1 cm; **D,E**, 200 μm; **F–L**, 20 μm.

Some mutants have defective reproductivity and there is a very small amount of flowers. It would be useful to treat samples with different hormones or different concentrations of the same hormone separately to compare the responses on one plate. Therefore, we developed an excised pistil culture system. We removed or uncovered the outer organs of Arabidopsis flowers, and laid the pistils horizontally on the medium. Since the outer organs had been removed, the naked pistils could absorb water, nutrients, and hormones through the surface cells and their absorption efficiency was much enhanced. In this system, the pistils were prone to losing water and becoming stressed. Therefore, we developed a simple system to maintain nearly 100% humidity by placing the small dish containing medium and pistils inside a larger dish containing a piece of wet sterilized filter paper (**Figure 3A**, detailed description in section “Materials and Methods”).

Like in the experiments on excised flowers, we chose stage 8 and stage 9 pistils to initiate the pistil culture. We found that the excised pistils grew and developed normally from stage 8 (**Figures 3B,D**) or early stage 9 in the culture system. After 7 days of culture (**Figures 3C,E**), the ovules from 38.3% pistils had developed to stage 11 from stage 8 (26% in early stage 11, 12.3% in middle stage 11, and 0% in late stage 11), and the outer and inner integuments had started to encase the nucellus (**Figures 3F–H**). Analyses of the *pKNU:KNU-Venus* marker line showed that the megaspore mother cells differentiated normally, meiosis occurred, and then a functional megaspore formed and began to undergo mitosis (**Figures 3I–L**). The ovules from 80% pistils developed past late stage 11 from stage 9 in this culture system (**Figure 3L** and Supplementary Figures S4A–F). These results indicated that the pistils were able to grow normally in the culture system. We also demonstrated that the later the stage of the excised pistils at the start of culture, the better their growth in the culture system. As expected, the ovules in excised flowers grew and developed better than did the ovules in excised pistils.

### High Efficiency of Hormone Treatments in Excised Flower/Pistil Culture System

**FIGURE 4:**
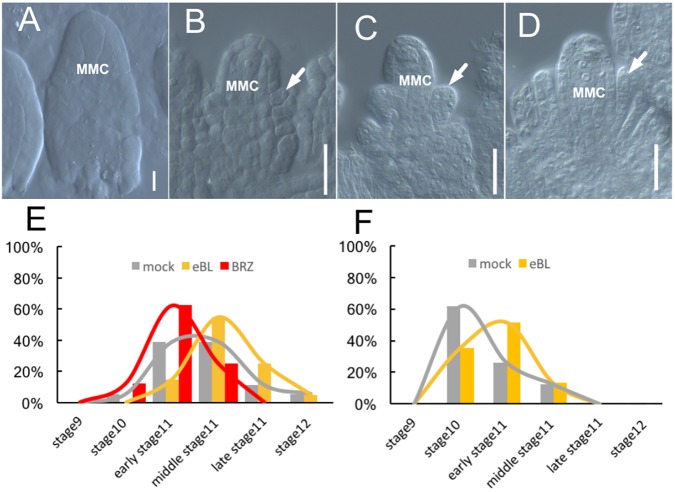
Ovule development after BR treatment in culture systems. **(A–D)** The ovules develop inside cultured Col-0 flowers after 7 days treatment by 1 × 10^-8^ mol/L eBL treatment, the ovules at stage10 **(A)**, at early stage 11 **(B)**, middle stage11 **(C)**, and late stage 11 **(D)**. White arrows in **(B–D)** indicate the integuments. MMC, macrospore mother cell. Bar, 20um. **(E)** The ovules inside the cultured Col-0 flowers develop to different ovule developmental stages after treating by 1 × 10^-8^ mol/L eBL and 1 × 10^-6^ mol/L BRZ. **(F)** The ovules inside the cultured *pKNU:KNU-Venus* pistils develop to different ovule developmental stages after 7 days treatment by 1 × 10^-8^ mol/L eBL.

In a preliminary experiment, we added 1 × 10^-6^, 1 × 10^-7^, and 1 × 10^-8^mol/L eBL to our medium to culture excised flowers in stage 8. The aim of this experiment was to detect which concentration of eBL was most suitable for further experiments. After 7 days of treatment, the flowers treated with 1 × 10^-8^mol/L eBL showed the best ovule development (Supplementary Figure S5). Next, we conducted three independent experiments using 1 × 10^-8^mol/L eBL. Observations of ovule and stigma development, especially integument growth, indicated that the eBL treatment enhanced the development of excised flowers and ovules (**Figure 4** and Supplementary Figures S6A,B). For example, after the eBL treatment, the ovules from all flowers pasted stage 10 (**Figure 4A**), 15% were in early stage 11 (**Figure 4B**), 55% were in middle stage 11 (**Figure 4C**), 25% were in late stage 11 (**Figure 4D**), and 5% were in stage 12. In the control, the ovules of 5.56% flowers were in stage 10, 38.89% were in early stage 11, 38.88% were in middle stage 11, 11.11% were in late stage11, and 5.56% were in stage 12 (**Figure 4E** and Supplementary Figures S6A,B).

When we conducted three independent experiments using 1 × 10^-6^mol/L BRZ, a brassinosteroid synthesis inhibitor, to our medium, the development of flowers and ovules was delayed compared with those in the control (no BRZ) after 7 days of growth (**Figure 4E** and Supplementary Figures S6A,B). For example, the ovules from 12.5% flowers were in stage 10, 62.5% were in early stage 11, 25% were in middle stage 11, and none was in late stage 11 or stage 12 (**Figure 4E**). These results illustrated that BRZ inhibited the development of excised flowers and ovules.

We also directly dropped 1 × 10^-8^ mol/L eBL onto *pKNU:KNU-Venus* pistils (which grow more slowly than pistils of *Col-0*) in stage 8 using a pipette. The results of three independent experiments further demonstrated that eBL promoted ovule development (**Figure 4F** and Supplementary Figures S6C,D). For example, in the eBL treatment group, the ovules from 35.45% pistils were in stage 10, 51.4% were in early stage 11, 13.15% were in mid-stage 11, and none was at late stage 11 or stage 12 (**Figure 4F**). In the control, the ovules from 61.7% pistils were in stage 10, 26% were in early stage 11, 12.3% were in mid-stage 11, and none was at late stage 11 or stage 12 (**Figure 4F**). These results not only demonstrated that eBL promoted ovule development, but also showed that our culture and treatment systems were highly efficient for the application of hormone and chemical treatments.

## Discussion

Investigating the regulatory mechanism of ovule development has both scientific significance and wide potential applications. Developmental, hormonal, and environmental signals can affect ovule development. In Arabidopsis, the ovule is embedded inside multiple layers of tissues and difficult to observe and treat in living plants. The spraying method is well known and has been widely used, although differences in evaporation ratios can introduce varied results and one spraying treatment may affect many inflorescence apexes. Our method to treat ovules in living plants is stable and repeatable, and provides another option.

Some reports have described the *in vitro* culture of fertilized ovules or developing ovules for a short period beginning from a late stage (ovules in stage 12 or later) (Sauer and Friml, 2004). However, it is more difficult to study ovule development in early stage because the ovules are too small to separate and are severely damaged by dissection. We tested some other species including *Brassica napus*, but the flowers, pistils, and ovules of larger species in early developmental stages were still too small to be separated and cultured easily (data not shown).

Most of the ovules in the excised flowers and separated pistils in our culture system were able to develop to the late flower developmental stage 11, and some could develop to stage 12. Our methods enhanced the efficiency of hormone and chemical treatments, confirming their suitability for use in research on ovule development, especially before stage 12 (since a few ovules progress to stage 12). Analyses of the ovule morphology of *Col-0* and the fluorescence signals from *pKNU:KNU-Venus* demonstrated that the ovules grown in our culture system were living and functional. The results of several independent experiments confirmed that eBL promoted ovule development. Although the ratios of ovules in particular developmental stages varied among experimental replicates, the trends were consistent (**Figures 1W**, **4E,F** and Supplementary Figures S6A–D). We will keep making improvements to our system until most of the ovules in excised flowers and pistils can complete all developmental stages. At present, our system is limited to studies on late ovule development. In our method, the pistils and flowers are dissected from the plant inside a clean hood. After 10 days, most of the ovules in excised flowers cannot continue to grow, even when the plate is uncontaminated. Also, most of the ovules cannot keep growing when they are moved to new medium.

In our method, the stalks of excised flowers are pushed into the medium without being sterilized by ethanol, and are then grown in normal conditions (see section “Materials and Methods”). Because ethanol sterilization can negatively affect plant tissues, we prefer to omit this step. We found that less than 20% of the plates were contaminated after 8–10 days. Eight days is long enough to observe ovule growth and development to stage 12. The low contamination ratio after 7 days might be because the flower buds in stage 8 are small (200 μm) and are located in the center of the inflorescence apex, where they are surrounded by bigger buds.

We also removed outer flower organs and separated pistils to transfer onto growth medium. The excised pistils were laid horizontally instead of being pushed vertically into the medium to reduce water loss. Establishing 100% humidity conditions (see section “Materials and Methods”) enhanced the survival ratio of the cut pistils. Sometimes the pistil was damaged during the dissection process, but this did not greatly affect ovule growth. Since the pistils were separated from the flower buds inside a clean hood, the contamination ratio was lower than that of excised flowers.

It is much easier to work with excised flowers than with excised pistils. Comparing the flower system and the pistil system, the former has the simplest protocol, while the latter has the lowest contamination rate and the highest efficiency for saving materials. Also, the pistil system is suitable for parallel treatments with different hormones and chemicals, because different solutions can be dropped onto single pistils on the same plate. In contrast, only one treatment can be applied to each plate in the flower system because the chemical/hormone is added to the medium. The culture systems and treatment for excised flower/pistil are much more efficient than treatment in living plant system (even our improved system) because the developmental stages can be accurately identified and the flowers in the same developmental stages can be used for different treatments. It would be much helpful for saving plant materials especially for mutants with reduced reproduction. Besides hormones or chemicals, environmental factors (e.g., temperature) could easily be tested in our system. Thus, our system has wide potential applications.

We tested excised flowers and pistils at different developmental stages in our system. Basically, the later the starting stage, the better the ovule growth in our culture systems. For research on earlier ovule development, we start from stage 8 or early stage 9. We prefer to start from stage 8 when studying the regulation of ovule primordial initiation. Ovule development is not completely synchronized in a single flower/pistil, but the developmental stage of most ovules will be in 1–2 contiguous stages. It would be a main stage of ovule development in the same flower/pistil. We can statistically analyze the proportions of ovule developmental stages through observing and calculating each ovule or each flower/pistil. The result is accurate for checking every single ovule, but it takes long time and will be difficult to handle. The result will be inaccurate if some ovules are missed or damaged during the operation. We prefer to check the main stage of ovule development in the same flower/pistil for statistical analysis. These analyses will be helpful to enhance the maneuverability and speed of experiments and for planning large-scale observations and screening. The statistical analysis will be modified if we study some mutants with unsynchronized ovule development in the same flower/pistil.

Most previously reported ovule culture systems result in developmental aberrations in varying frequencies (Kumlehn et al., 1997; Sauer and Friml, 2004). Because flowers and pistils are cultured in our systems, the ovules grow normally and do not show severe aberrations. We observed that the excised flowers accumulated anthocyanins on their surface and on the pistil. A proportion of ovules were able to develop to later stages, while some stopped developing. There were fewer ovule primordia in cultured flowers than in flowers of living Arabidopsis plants because of the artificial environment, but it did not influence the results since we had appropriate controls.

We have developed a treatment system for living plants and two culture and treatment systems for excised flowers/pistils. These systems will be useful for research on ovule development and the hormonal regulation of this process. Our systems are simple and easy to operate, and will be useful for further studies on ovule development including hormonal regulation, hormone crosstalk, chemical screening, and even the effects of stresses.

## Author Contributions

W-HL designed the study, wrote and modified the manuscript, and acquired funding. B-FL and S-XY performed the experiments and helped to write the manuscript. L-QH acquired DIC images of pistils. NZ helped to acquire confocal microscope images. Y-JZ modified the figures and helped to write the manuscript. LX helped to organize the results and the manuscript. All authors agreed to be accountable for the content of this paper.

## Conflict of Interest Statement

The authors declare that the research was conducted in the absence of any commercial or financial relationships that could be construed as a potential conflict of interest.
