# Supplementary material for: Simple Culture Methods and Treatment to Study Hormonal Regulation of Ovule Development
Source: Front Plant Sci. 2018 Jun 18;9:784. doi: 10.3389/fpls.2018.00784 (PMC6015908; doi:10.3389/fpls.2018.00784)
Supplement: Supplementary file 1 [file Image_1.PDF]

Li and Yu et al, Supplementary figure 1

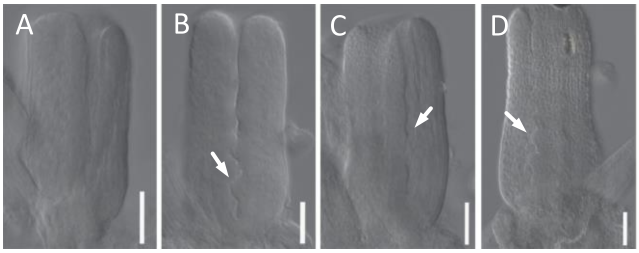

Figure S1 Ovule development inside cultured pistils on 1/2 MS medium.
(A-D) Ovule development from stage 8 (A-B) and stage 9 (C-D) on 1/2 MS medium after 7 days growth. Bar=20um. White arrows indicate the initiated ovules (B-D).

Li and Yu et al, Supplementary figure 2

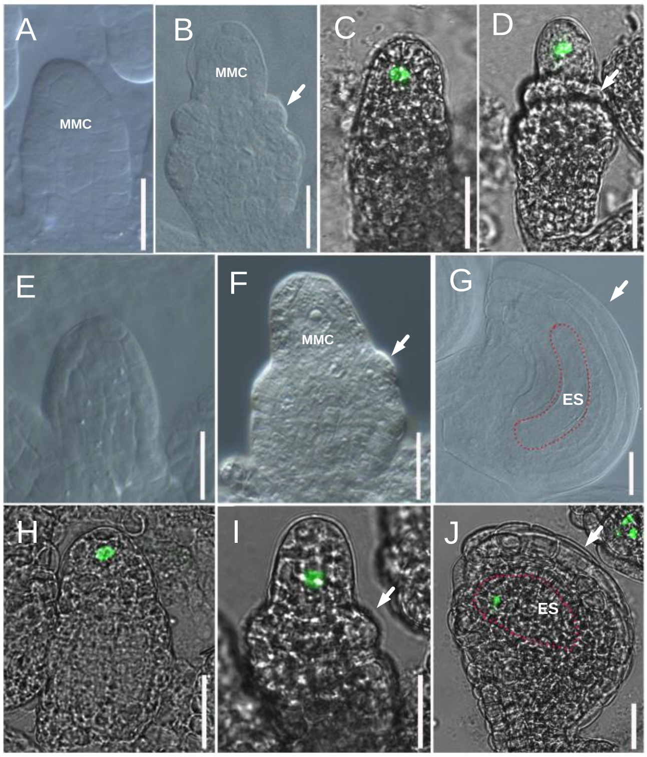

Figure S2 Ovule development inside cultured flowers on 1/2 MS medium.
(A-D) Ovules in cultured flowers from stage 8 develop to stage 10 (A, C) and stage 11 (B, D). (E-J) Ovules in cultured flowers from stage 9 develop to stage 10 (E, H), stage 11 (F, I), and stage 12 (G, J). White arrows in (B, D, F, G, I and J) indicate the integuments, and the areas highlighted by the dotted line indicate the embryo sac in (G) and (J). MMC, macrospore mother cell. ES: embryo sac. Bars = 20 μm.

Li and Yu et al, Supplementary figure 3

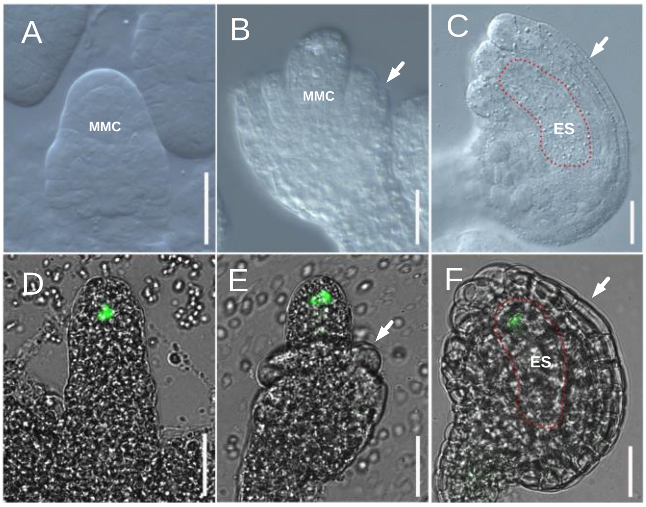

Figure S3 Ovule development inside cultured flowers on culture medium.
(A-F) Ovules inside cultured flowers from stage 9 develop to stage 10 (A, D), stage 11 (B, E), and stage 12 (C, F). White arrows in (B), (C), (E), and (F) indicate the integuments, and the areas highlighted by the dotted line indicate the embryo sac in (C) and (F). MMC, macrospore mother cell. ES: embryo sac. Bars = 20 μm.

Li and Yu et al, Supplementary figure 4

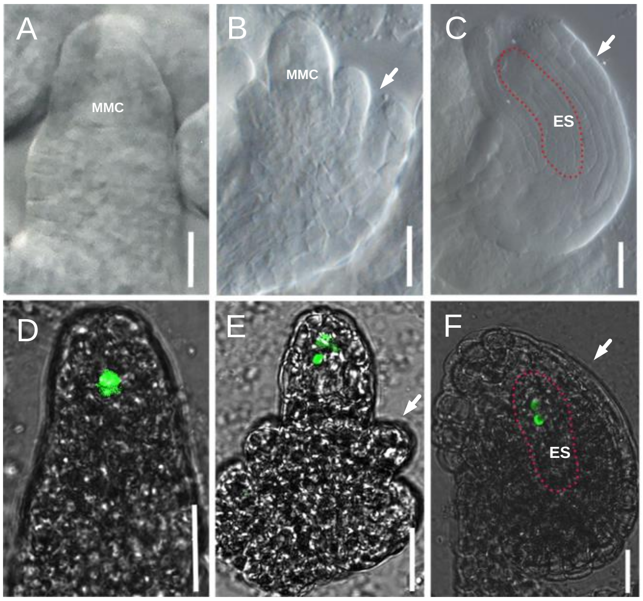

Figure S4 Ovule development inside cultured pistils on culture medium.
(A-F) Ovules inside cultured pistils from stage 9 to stage 10 (A, D), stage 11 (B, E), and stage 12 (C, F). White arrows in (B), (C), and (E) indicate the inner and outer integuments, and the areas highlighted by the dotted line indicate the embryo sac in (C) and (F). MMC, macrospore mother cell. ES: embryo sac. Bars = 20 µm.

Li and Yu et al, Supplementary figure 5

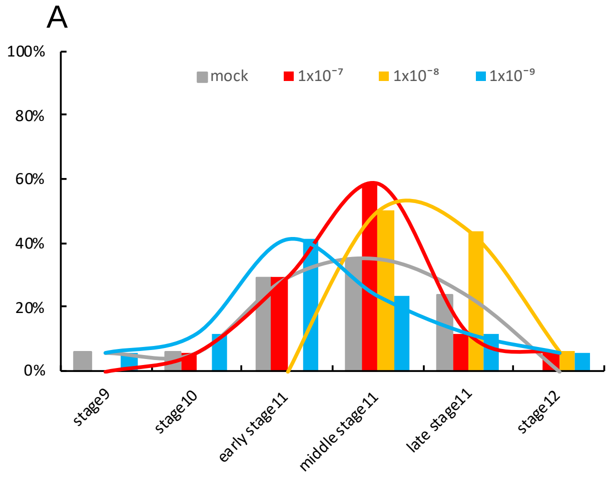

Figure S5 The effect of different concentration of BR treatment on ovule development in excised flowers.

Li and Yu et al, Supplementary figure 6

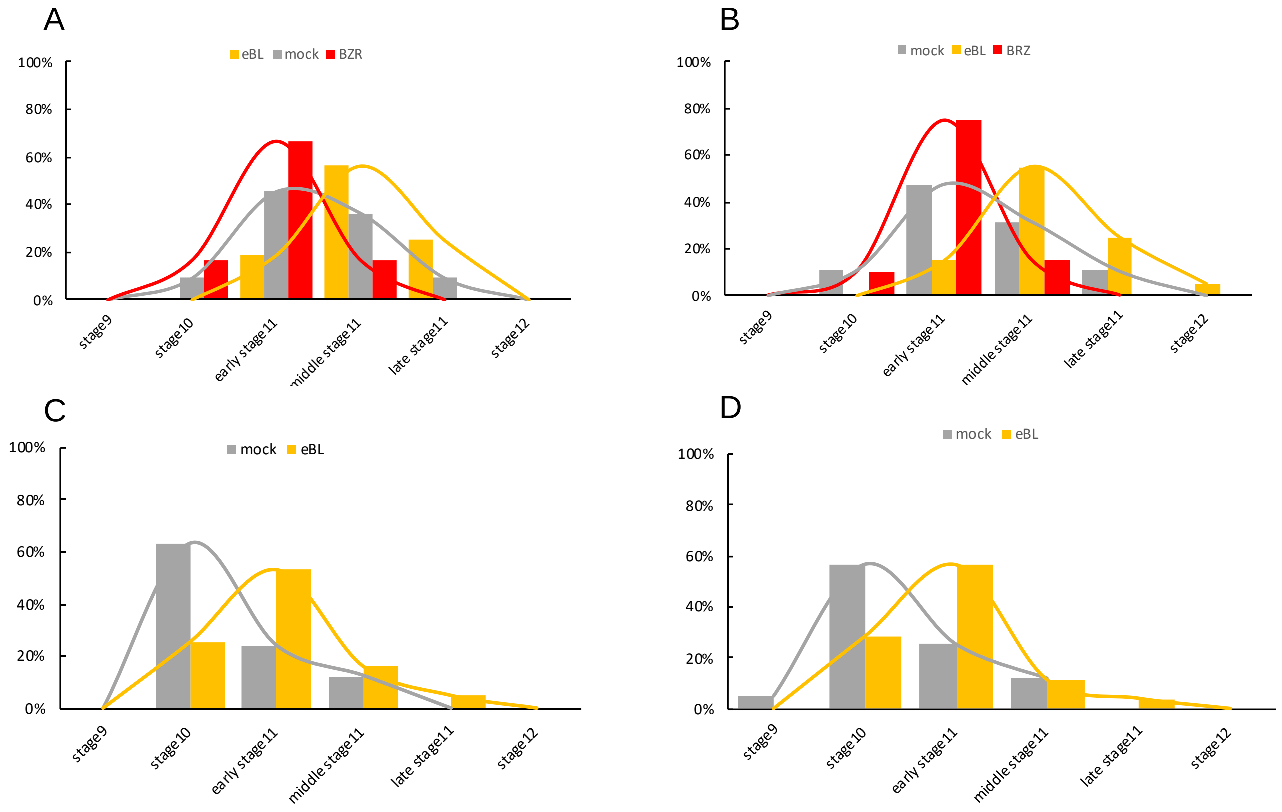

Figure S6 Ovule development after BR treatment in excised flowers/pistils (another twice independent experiments).

(A-B) The ovules inside the cultured Col-0 flowers develop to different ovule developmental stages after 7 days treatment by 1×10^{-8} mol/L eBL and 1×10^{-6} mol/L BRZ. (C-D) The ovules inside the cultured *pKNU:KNU-Venus* pistils develop to different ovule developmental stages after 7 days treatment by 1×10^{-8} mol/L eBL.
